# Phylogenetic Variation of *Tri1* Gene and Development of PCR–RFLP Analysis for the Identification of NX Genotypes in *Fusarium graminearum* Species Complex

**DOI:** 10.3390/toxins15120692

**Published:** 2023-12-08

**Authors:** Meiling Gao, Mengyuan Zhang, Jiahui Zhang, Xianli Yang, Mohamed F. Abdallah, Jianhua Wang

**Affiliations:** 1 Institute for Agro-Food Standards and Testing Technology, Ministry of Agriculture, Shanghai Academy of Agricultural Sciences, 1000 Jinqi Road, Shanghai 201403, China; gaoml@dlpu.edu.cn (M.G.); m210300781@st.shou.edu.cn (M.Z.); jiahuizhang1224@163.com (J.Z.); yangxianli@saas.sh.cn (X.Y.); 2 National Engineering Research Center of Seafood, School of Food Science and Technology, Dalian Polytechnic University, Dalian 116034, China; 3 Department of Food Technology, Safety and Health, Faculty of Bioscience Engineering, Ghent University, Coupure Links 653, 9000 Gent, Belgium; mfathiabdallah@gmail.com; 4 Department of Forensic Medicine and Toxicology, Faculty of Veterinary Medicine, Assiut University, Assiut 71515, Egypt

**Keywords:** *Fusarium graminearum* species complex, trichothecenes, PCR-RFLP, NX toxins

## Abstract

NX toxins have been described as a novel group of type A trichothecenes produced by members of the *Fusarium graminearum* species complex (FGSC). Differences in structure between NX toxins and the common type B trichothecenes arise from functional variation in the trichothecene biosynthetic enzyme Tri1 in the FGSC. The identified highly conserved changes in the *Tri1* gene can be used to develop specific PCR-based assays to identify the NX-producing strains. In this study, the sequences of the *Tri1* gene from type B trichothecene- and NX-producing strains were analyzed to identify DNA polymorphisms between the two different kinds of trichothecene producers. Four sets of Polymerase chain reaction–restriction fragment length polymorphism (PCR-RFLP) methods were successfully developed to distinguish the common type B trichothecene producers and NX producers within FGSC. These promising diagnostic methods can be used for high-throughput genotype detection of *Fusarium* strains as a step forward for crop disease management and mycotoxin control in agriculture. Additionally, it was found that the *Tri1* gene phylogeny differs from the species phylogeny, which is consistent with the previous studies.

## 1. Introduction

**Figure 1:**
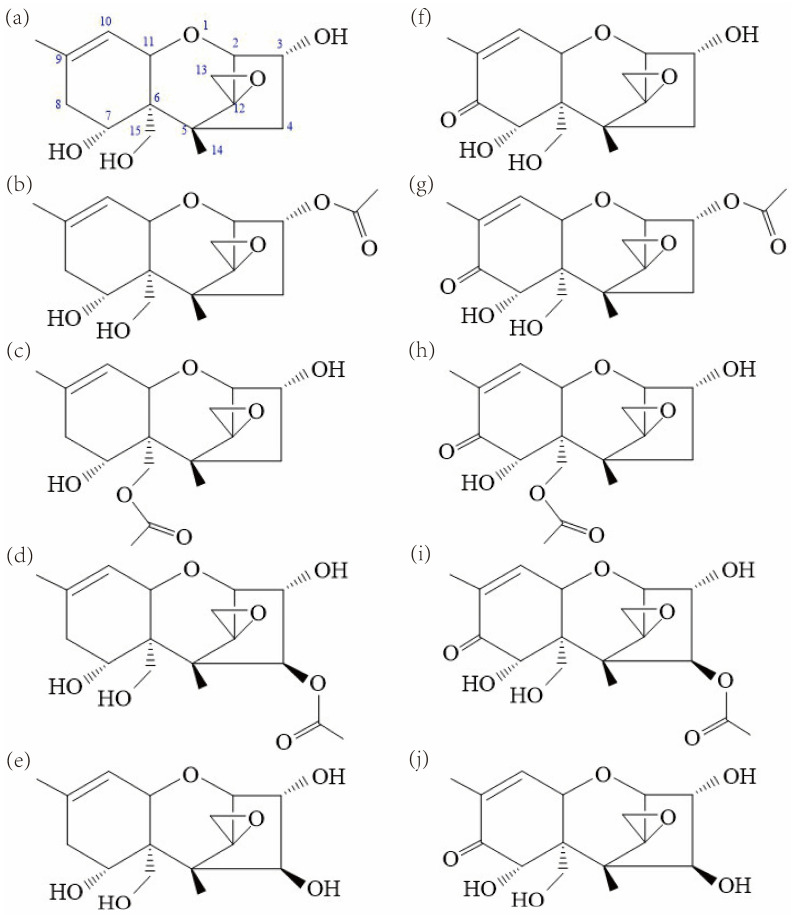
Structures of (**a**) NX-3, (**b**) NX-2, (**c**) NX-4, (**d**) NX-5, (**e**) NX-6, (**f**) DON, (**g**) 3ADON, (**h**) 15ADON, (**i**) 4ANIV and (**j**) NIV produced by FGSC. Both NX-5 and NX-6 are proposed and have not been identified in nature.

Members of the *Fusarium graminearum* species complex (FGSC) are considered the main causative agents of *Fusarium* head blight (FHB) in wheat, barley, oats, and other small cereal grain crops all over the world [1]. Significant economic losses in cereal production have already been documented in North America [2,3,4], China [5,6,7], and other regions of the world. Over the last two decades, investigations revealed that the FGSC consists of at least 16 phylogenetically distinct species with varying geographical distribution, pathogenicity, and mycotoxin profiles [8,9,10]. *Fusarium* toxins are frequent contaminants of cereals worldwide, with trichothecenes (Type A and B) as the predominant toxins [11]. Trichothecenes inhibit eukaryotic protein synthesis, and they are responsible for emetic, anorexic, immunosuppressive, and even death in severe cases of exposure [12,13,14,15]. Additionally, trichothecenes can act as virulence factors and facilitate tissue colonization in specific plant hosts [16,17,18]. Therefore, the impacts of *Fusarium* mycotoxins on food safety and human health have recently aroused considerable public concern [5]. Type B trichothecenes have a 7-hydroxy, 8-keto-trichothecene core structure and are the most frequently occurring mycotoxins in cereal crops worldwide [19]. Deoxynivalenol (DON) along with its acetylated derivatives, 3-acetyldeoxynivalenol (3ADON) and 15-acetyldeoxynivalenol (15ADON), as well as nivalenol (NIV) along with its acetylated derivative 4-acetylnivalenol (4ANIV), are the most common type B trichothecenes produced by the FGSC (Figure 1). From trichothecene production profiles, the FGSC strains are subdivided into three different genotypes/chemotypes: (1) 3ADON genotype/chemotype, which produces DON and 3ADON; (2) 15ADON genotype/chemotype, which produces DON and 15ADON; (3) and NIV genotype/chemotype which produces NIV and 4ANIV [20,21,22,23,24]. Previously, it was known that the FGSC strains typically produce one of the three strain-specific profiles of type B trichothecenes. However, in 2014, new isolates of the FGSC were isolated, which can produce a novel group of type A trichothecenes called NX toxins (NX-2, NX-3, and NX-4), as shown in Figure 1 [25,26,27,28].

NX toxins are similar in structure to common type B trichothecenes. The NX-2 and NX-4 toxins are the acetylated form of NX-3 at C-3 and C-15, respectively. In comparison with the common type B trichothecenes (DON, 3ADON, and 15ADON), the novel type A trichothecenes NX-3, NX-2, and NX-4 lack the keto group at C-8, respectively (Figure 1). The obtained genetic analysis revealed a different *Tri1* allele in the NX producers, which was verified to be responsible for the specific oxidation at C-7 alone [26]. Hence, the structural variations at C-8 between these two groups of trichothecenes result from the functional diversity of trichothecene biosynthetic enzyme Tri1 [26].

In recent decades, tremendous progress has been made toward genotyping analysis of type B trichothecene producers. Different PCR-based genotyping techniques have been developed and extensively used for genotyping analysis of FGSC populations. All these genotyping techniques target trichothecene biosynthesis genes, such as *Tri13*, *Tri3*, *Tri12*, and *Tri7*, and PCR assays arose from the *Tri* gene polymorphisms of the *Fusarium* strains with different trichothecene profiles. Molecular genetic assays allow high-throughput screening of many field strains [21,23,29,30,31,32,33,34,35]. Such tools, with single or multiplex PCR, were used in different regions in the world, including Asia [24], Europe [36], and America [37,38], proving their efficiency and reliability in detecting the DON/NIV chemotypes and their acetylated forms [34]. However, accurate high-throughput molecular diagnostic methods for NX producers are yet to be developed.

Therefore, the current work aimed to (1) investigate the polymorphism of *Tri1* gene in the NX and common type B trichothecene producers; (2) develop a NX-trichothecene genotyping method based on *Tri1* gene polymorphism; and (3) discuss the potential trichothecene productivities of the NX strains and infer the potential biosynthetic pathway of the NX toxins.

## 2. Results

### 2.1. Phylogenetic Analysis of Tri1 Gene

A total of 379 *Tri1* gene DNA sequences were analyzed by using multiple sequence alignment assays and the duplicate sequences were removed. After that, 103 representative *Fusarium Tri1* gene sequences (87 from common type B trichothecene producers, 6 from NX producers, and 10 from T-2 producers) were identified and used for further phylogenetic relationship analysis.

**Figure 2:**
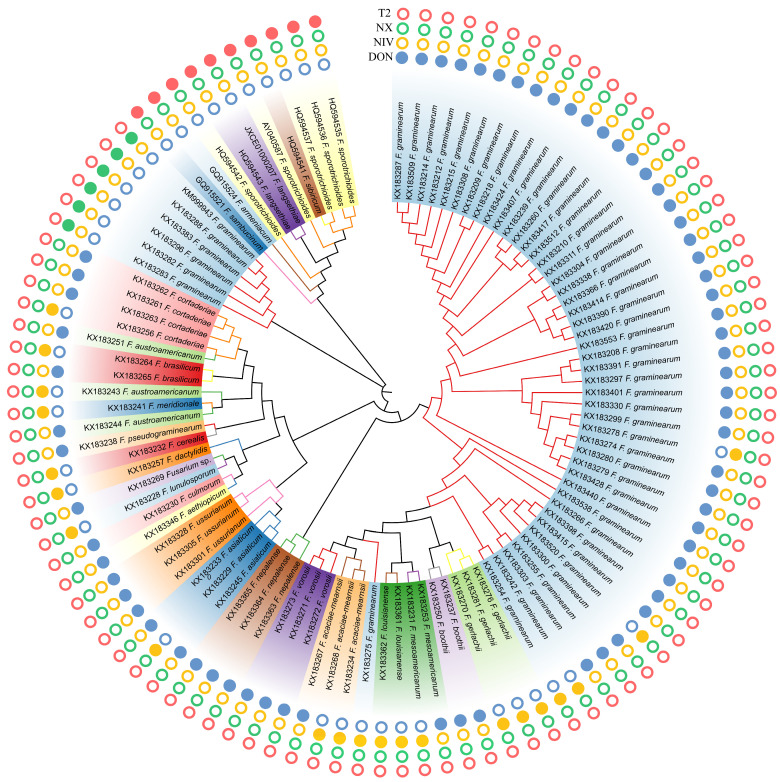
Neighbor-joining bootstrapped phylogeny of *Tri1* gene DNA sequences (*N* = 103) from common type B trichothecene-producing strains, NX-, and T-2 producers of *Fusarium*. Phylogeny was inferred using the Kimura 2-parameter model of nucleotide substitution with a Gamma parameter to account for the heterogeneity rate. *Fusarium* species and their trichothecene toxin chemotypes are indicated. A solid circle indicates that the strain can produce T-2, NIV, DON, or NX toxins, while an empty circle means the strain cannot produce the corresponding metabolites.

The *Tri1* gene phylogeny resolved type B trichothecene, NX, and T-2 strains into three different major clades (Figure 2). However, the evolution of *Tri* genes does not always correlate with the evolutionary history of *Fusarium* species divergence according to the inferred *Tri1* phylogeny. For instance, both *F. graminearum* and *F. sporotrichioides* were polyphyletic in the *Tri1* tree, and high divergence of NX *F. graminearum* strains from the type B-trichothecene producers was observed according to the inferred topological structure of *Tri1* genes. Although the placement of the divergent NX clade was the most obvious conflict between the *Tri1* tree and the recognized species phylogeny, other conflicts were also observed. For example, the two newly characterized monophyletic species, *F. austroamericanum* and *F. dactylidis*, and the four early diverging species, including *F. cerealis*, *F. culmorum*, *F. lunulosporum*, and *F. pseudograminearum*, were all nested within the FGSC. In the current investigation, *Tri1* phylogeny is not always correlated to species phylogeny, which is consistent with the conclusion made by other studies [23,39,40].

### 2.2. Polymorphism of Tri1 Gene CDS Sequences

To assess the diversity of *Tri1* coding sequences in NX- and type B-trichothecene-producing strains, the polymorphism of *Tri1* coding sequences (CDSs) was analyzed. In total, 93 representative *Tri1*-CDSs were analyzed, excluding ten T-2 producing strains. In general, the *Tri1* gene of the 93 strains varied from 1753 to 1759 bp in total length with four introns, and the coding region is 1539 nucleotides. After the duplicate sequences were removed, 64 CDS sequences remained, and the identity of these coding sequences ranged from 96.23% to 99.94%.

### 2.3. Specific Amino Acid Sites for NX Strains

**Figure 3:**
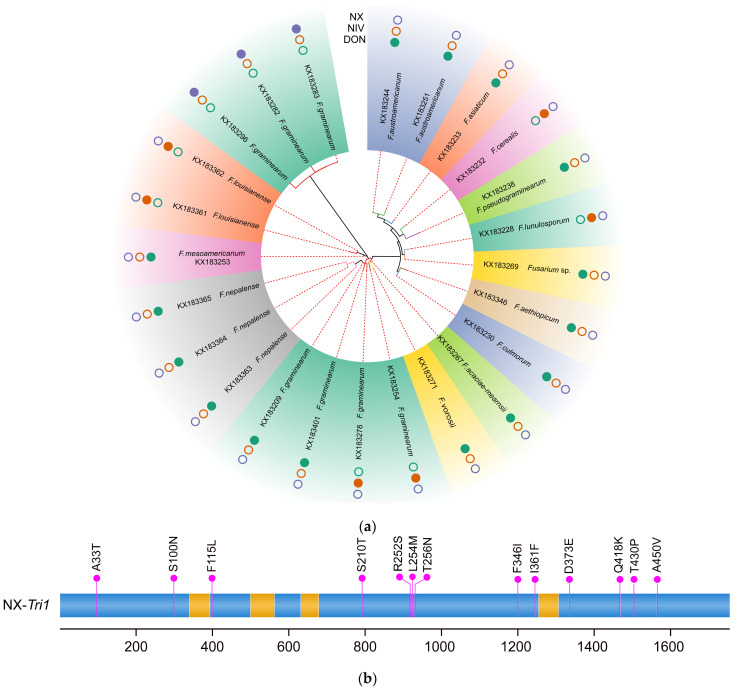
(**a**) Phylogeny of Tri1 amino acid sequences inferred with 24 representative strains. A solid circle indicates that the strain can produce NX, NIV, or DON toxins, while an empty circle means the strain cannot produce the corresponding metabolites. (**b**) Amino acid differences between the *Tri1* gene products of NX strains and common type B trichothecene producers. The amino acids specific to NX producers are indicated above the panel; for example, “A33T” indicates that “T” is specific to NX producers, whereas “A” is specific to type B trichothecene producers. The nucleotides and amino acids are numbered as referred to in the sequence of NX strain 02-264.

Apart from the *Tri1* gene of the T-2 toxin producers, 24 representative protein sequences were identified using the deduced amino acid sequences of the other 366 *Tri1* genes. As shown in Figure 3, the phylogeny inferred from predicted Tri1 amino acid sequences is quite similar in topology to the *Tri1* gene tree and also showed strong evidence for the divergence of NX strains from the type B trichothecene strains. Consequently, 13 amino acid differences were identified between the *Tri1* gene products of common type B strains and NX producers (Figure 3). Previously, by comparing the analysis of amino-acid sequences inferred based on the predicted *Tri1* coding sequence of PH-1 (15ADON producer) and 20 NX producers, 14 amino acid differences between the two kinds of trichothecene producers were identified by Varga et al. [26]. All the 13 amino acids identified in our work are entirely consistent with the results provided by Varga et al. [26]. The amino acid identified by Varga et al. [26] at position 363 (Valine/Isoleucine) was neither specific to type B nor NX producers, with isoleucine only found in NX producers. In contrast, both isoleucine and valine were identified in type B trichothecene producers. As shown in Figure 3, the 13 different amino acids between the two groups of trichothecene producers appeared to be distributed randomly in the *Tri1* gene. According to the analysis, a conclusion can be made that there are no more than 13 amino acid differences that determined the production of NX toxin or type B trichothecenes in FGSC.

### 2.4. PCR-RFLP Based Genotyping Analysis of NX Producers

To investigate the reliability of the PCR-based analysis for identifying NX-producing FGSC strains, 30 strains collected from different areas were tested. The results showed that primer pairs Tri1F/R1 and Tri1F/R2 amplified a specific fragment approximately 439 bp and 899 bp, respectively, with all the tested FGSC strains.

**Figure 4:**
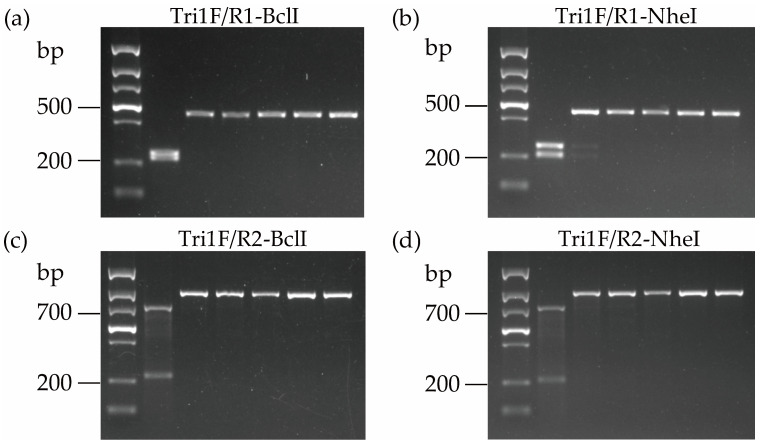
The electrophoresis of the PCR products was digested by the two endonucleases. (**a**) PCR fragments amplified with primers Tri1F/R1 and digested by BclI; (**b**) PCR fragments amplified with primers Tri1F/R1 and digested by NheI; (**c**) PCR fragments amplified with primers Tri1F/R2 and digested by BclI; (**d**) PCR fragments amplified with primers Tri1F/R2 and digested by NheI. Lane M, DNA marker; Lane 1, NX producer; Lanes 2–6, type B trichothecene-producing strains.

As depicted in Figure 4, after digestion, the PCR products amplified via NX-Tri1F/R1 from NX producers yielded two restriction fragments (198 bp and 241 bp for NheI, and 208 bp and 231 bp for BclI), but only a 439 bp fragment could be recovered from common type B trichothecene producers. The digested PCR products from NX producers cannot be separated well on an agarose gel after BclI digestion due to a 23 bp difference. However, the band size is distinct from type B trichothecene producers (Figure 4). Similarly, two restriction fragments with noticeable size differences (198 bp and 701 bp for NheI, 208 bp and 691 bp for BclI) were produced for the PCR products amplified by the primer pair NX-Tri1F/R2 from the NX producers, while only a single 899 bp fragment exists for the type B trichothecene producers after digestion (Figure 4). The distinct DNA fragment numbers and sizes from the NX and type B trichothecene-producing strains generated via the PCR-RFLP approach demonstrated that this method can differentiate these two trichothecene-producer groups in FGSC. With the combination of different primer pairs and restriction endonucleases, these four diagnostic methods are proposed for identifying NX-producing strains based on the *Tir1* gene.

### 2.5. Validation of the Previously Developed ApoI-Based PCR-RFLP Genotyping Analysis of NX Producers

Recently, a PCR-RFLP-based diagnostic test was developed and validated for the NX-producing strains relying on polymorphisms in the *Tri1* gene [28]. Portions of the *Tri1* gene were amplified using primers Tri1F (5′-ATGGCTCTCATCACCAG-3′) and Tri1R (5′-CAATTCCAATCGCAGACAA-3′), and an amplicon of approximately 1740 bp was generated from both type B and NX trichothecene-producing FGSC strains. After digestion with restriction endonuclease ApoI, two restriction fragments of 888 bp and 851 bp were produced for strains with 3ADON, 15ADON, and NIV chemotypes and three fragments of 407 bp, 482 bp, and 851 bp were generated for the NX strains. Kelly et al. employed this technique to analyze FGSC strains from 19 different countries to determine the geographic distribution of the NX strains [27].

**Figure 5:**
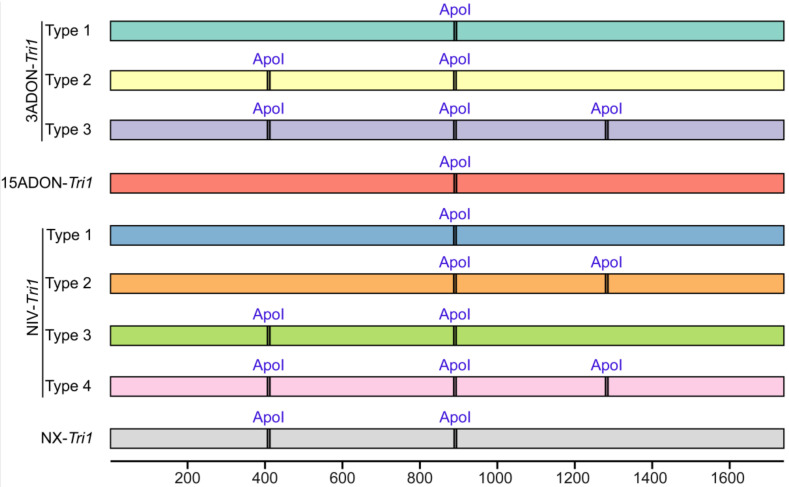
Diagrammatic presentations of the ApoI sites of *Tri1* gene DNA sequences amplified with primer pair Tri1F/R [26] from *Fusarium* strains with common type B trichothecene and NX chemotypes. It is an in silico analysis from published sequences, and the nucleotides are numbered as referred to in the sequence of NX strain 02-264.

To assess the reliability of the Tri1-ApoI-based genotyping method developed by Liang et al. [28], the ApoI sites of 93 *Tri1* gene amplicons with primer pair Tri1F/R were analyzed. As shown in Figure 5, the expected fragment size amplified with primer pair Tri1F/R is about 1740 bp in length. Three ApoI enzyme site types were identified for 3ADON producers, and four types were identified for NIV producers, while only one type was found for 15ADON producers. Exactly the same band sizes and numbers would be obtained with 3ADON-Tri1 type 2, NIV-Tri1 type 3, and NX-Tri1 sequences. Moreover, similar products were observed from NIV-Tri1 type 2 and NX-Tri1 strains after ApoI restriction enzyme digestion, which probably can lead to misinterpretation during electrophoresis separation on agarose gel. So, in part at least, the Tri1-ApoI-based assay from Liang et al. [28] cannot effectively discriminate the NX strains from common type B trichothecene producers.

### 2.6. Potential Trichothecene Productivities and Proposed Biosynthetic Pathways of NX Toxins

**Figure 6:**
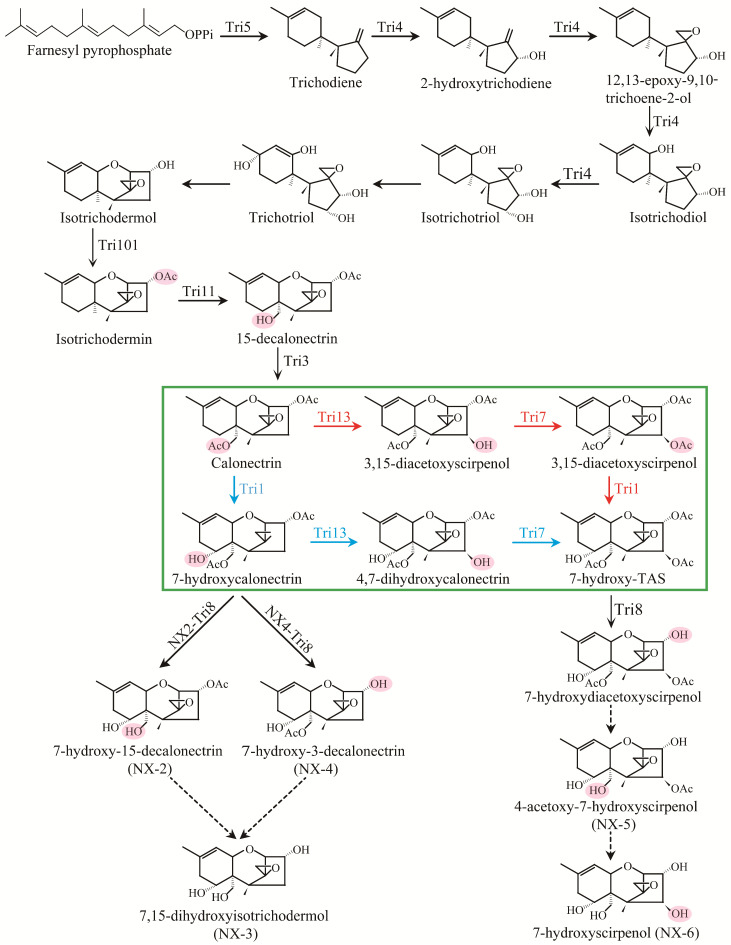
Proposed biosynthetic pathways of NX-trichothecenes in FGSC. Genes encoding an enzymatic step are identified near the arrow indicating the step. Dashed arrows indicate steps for which genes are unknown. The green box identifies the two alternative pathways of NX-5 and NX-6, respectively. The Tri13-Tri7-Tri1-Tri8 and Tri1-Tri13-Tri7-Tri8 pathways are marked in red and blue, respectively.

Although the biosynthesis molecular mechanisms of the NX toxins have not been fully revealed through experimental investigation, their possible biosynthetic pathways can be predicted based on the findings of T-2 and common type B trichothecenes discovered so far. As reported by Chen et al. [25], NX and type B trichothecene FGSC have similar trichothecene biosynthesis clusters. The hypothesized biosynthetic pathways of the NX toxins corresponding to DON, 3ADON, 15ADON, and T-2 toxins are depicted in Figure 6.

As previously reviewed in the literature, genetic variation within the *Tri1* gene dictated the specific synthesis of common type B trichothecenes and NX toxins in the FGSC strains [25]. Except for *Tri1*, the functions of other *Tri* gene alleles in the NX strains are well preserved compared to common type B trichothecene producers [25]. As a result, the majority of the genes in the biosynthesis pathway, such as *Tri3*, *Tri4*, *Tri5*, *Tri7*, *Tri8*, *Tri11*, *Tri13*, and *Tri101*, are strictly functionally conserved across the two forms of trichothecene. It is also known that in DON-producing strains, *Tri7* and *Tri13* genes are nonfunctional due to multiple insertions and deletions within their coding regions, which is not the case for NIV-producing strains [41,42,43]. During trichothecene biosynthesis, the reaction steps catalyzing farnesyl pyrophosphate to calonectrin are shared among *Fusarium* species. As shown in Figure 6, the biosynthesis of NX-2, NX-3, and NX-4 follow a Tri5-Tri4-Tri101-Tri11-Tri3-Tri1-Tri8 pathway, which is similar to DON and its acetylated derivatives (3ADON and 15ADON). For the biosynthesis of NX-5 and NX-6 toxins, there are two different pathways that both use calonectrin as a substrate: the Tri13-Tri7-Tri1-Tri8 pathway and the Tri1-Tri13-Tri7-Tri8 pathway. However, the precise biosynthetic pathway and regulatory mechanisms of NX toxins are unknown and worth further investigation.

## 3. Discussion

Previous studies revealed that the *Tri1* gene in *Fusarium* encodes a cytochrome P450 oxygenase catalyzing the hydroxylation of trichothecenes [44,45,46]. In *F. sporotrichioides*, *Tri1* encodes a cytochrome P450 monooxygenase enzyme that catalyzes the hydroxylation of trichothecenes at C-8, leading to the synthesis of type A trichothecenes, such as T-2 toxin and HT-2 [45,47]. However, two different situations were observed in the FGSC strains. The *Tri1* encodes a cytochrome P450 oxygenase needed for hydroxylation at both C-7 and C-8 positions, giving rise to type B trichothecenes, such as DON, NIV, and their acetylated forms [44]. On the other hand, in the NX FGSC strains, the enzyme encoded by *Tri1* responds to the hydroxylation at C-7 but not C-8 [25,26]. Consequently, allelic variants of *Tri1* are accountable for the structural variations in trichothecene toxins, and *Tri1* itself is a key gene in the biosynthesis of trichothecenes.

Even with the existing different hydroxylation activities, this *Tri1* gene may have the exact evolutionary origin within the FGSC [23]. Ward et al. concluded that the evolution of *Tri* genes has been maintained by balancing selection throughout the evolution of the fungi [23]. Kelly et al. conducted phylogenetic analyses of *Tri1* gene sequences to investigate the evolutionary origins of the NX producers [27]. Their results suggested that NX evolved more recently from a type B *Tri1* allele, possibly following the diversification of FGSC. Analysis of DNA polymorphism by Liang et al. [28] supports the hypothesis that NX-producing strains may be resolved from one of the more frequently represented populations reported by Gale et al. [48]. The fact that most of the NX strains characterized until now have core *Tri* clusters of the 3ADON type may indicate that the appearance of the NX-producing strains is connected to the recent expansion of the 3ADON population in North America [26]. The accumulation of nonsynonymous substitutions specific to the NX-2 clade indicated that the evolution of NX-2 may have been accompanied by significant changes in selective pressure on *Tri1* [27]. The main scenario behind the functional differences within the *Tri1* gene is still challenging to determine. However, these changes in the *Tri1* allele can be used to develop specific PCR assays for identifying NX-producing strains, such as amino acid sites 252S and 254M.

Trichothecene biosynthesis genes are commonly used as markers to detect the potential ability of a fungal strain of different *Fusarium* species to produce trichothecenes using specific PCR-based assays. In this study, a PCR-RFLP assay was developed based on the mutations that occurred between position 915 and 932 (“GCT CGC GAA CTA ATC ACT” to “GCT AGC GAA ATG ATC AAT”). These mutations in the *Tri1* coding region lead to the introduction of two new restriction enzyme cutting sites, G^∧^CTAGC and T^∧^GATCA (^∧^ indicates the cutting sites), which can be recognized by restriction endonuclease NheI and BclI, respectively. Despite the fact that just a few FGSC strains were tested using the current PCR-RFLP diagnosis approach, the results were highly confirmed by our phylogenetic analysis based on the 360 FGSC-*Tri1* gene sequences stated above.

The *Tri1* allele in NX-producing strains encodes a cytochrome P450 monooxygenase that hydroxylates only at the C-7 position. As concluded by Varga et al., the *Tri1* gene is the only functionally different *Tri* gene in the biosynthesis of trichothecene compared with the common type B trichothecene producers [26]. Therefore, a significant functional differentiation occurred in the *Tri1* gene within the FGSC strains, forming different trichothecene molecules. In this study, comparing the predicted Tri1 amino acid sequences from 327 common type B trichothecene and 39 NX *Fusarium* strains revealed 13 fixed differences between the two groups. Meanwhile, the functional divergence of the *Tri1* allele in the NX strains may arise from these mutations. These amino acid changes led to the development of our molecular approaches for specific identification of NX-determining mutations as part of pathogen monitoring efforts. Moreover, subsequent site-directed mutagenesis experiments targeting the amino acid residues identified as specific to NX producers should result in the determination of mutations responsible for NX biosynthesis.

Until now, two types of natural NX homologues, NX-2 and NX-3, have been identified in rice cultures and inoculated wheat ears. Compared with 3ADON, NX-2 lacks the keto functional group at the C-8 position characteristic for type B trichothecenes, and NX-3 is the deacetylated form of NX-2 at the C-3 position. In the heterologous complementary experiment by Varga et al. [26], five independent mutants were obtained for the PH-1 (15ADON producer) background carrying the NX-version of *Tri1* (IAWP48, 49, 84, 88, and 140). As expected, four of these five PH-1-derived mutants containing the *Tri1* from the NX strain WG-9 (IAWP48, 49, 84, 140) produced NX-2. Furthermore, a new toxin, NX-4, corresponding to 15ADON but lacking the keto functional group at the C-8 position, was identified in rice cultures [26]. However, it should be pointed out that NX-4 toxin-producing FGSC strains have not been identified, and this toxin is not known to occur naturally [25]. Likewise, it is still unclear whether fungal strains that produce the analogues of NIV and 4ANIV lacking the keto functional group at the C-8 position (described as NX-5 and NX-6 in Figure 1, respectively) naturally exist in the FGSC. As indicated in Figure 6, putative biosynthesis pathways for NX-4, NX-5, and NX-6 are hypothesized. More extensive screening is needed to reveal the possible existence of such strains.

Many studies can be conducted to investigate further the new emerging *Fusarium graminearum* population and its NX toxins. Firstly, determining the geographic distribution and characterizing the spatial and temporal dynamics of the NX-producing strains are crucial for disease management and control. For risk assessment, toxicity studies demonstrated that the NX-3 toxin can be considered equipotent to DON in the inhibitory effect of protein synthesis [26,49]. NX-2 shows a similar inhibitory effect on *Chlamydomonas reinhardtii* growth as 3ADON, as has been verified via toxicity assays [26]. In case the NX strains become more abundant, the NX toxins would be grouped with the common mycotoxin contaminants in cereals, posing an additional threat to food and feed safety. Secondly, it would be necessary to monitor whether the frequency of the NX strains is changing. To date, the NX producers have been recovered from different cereal crops, such as wheat, barley, and maize, indicating a wide range of plant hosts [26,27]. Notably, a high frequency (20%) of NX strains were recently reported in air samples collected in the USA [50]. The existence of the NX strains in airborne environmental samples will undoubtedly accelerate the expansion of the fungi. Due to international grain trade, there is a possibility of NX strains being introduced to different regions around the world. Although the overall frequency was low (2.8%), the NX-producing fungi were found during every sampling period [49]. Therefore, disease management and plant quarantine programs are needed to be more vigilant [23]. In this sense, the chemotype-specific PCR tests created in the current study offer a quick, precise, and direct genetic method for differentiating between strains that produce NX and common type B trichothecene. These tests could also be integrated into international monitoring programs to assess cereal contamination and characterize host-specific or biogeographic variations in chemotype distributions [23].

However, this group of new chemical substances should have unified and widely recognized scientific names, which will be conducive to implementing scientific research and academic exchanges. Therefore, an initiative for the naming/abbreviations of this group of new substances should be proposed. Hence, we suggest using NX, 3ANX, 15ANX, 4ANX, and 4HNX, similar to the naming of the common type B trichothecenes, such as DON, 3ADON, 15ADON, 4ANIV, and NIV, as described in several recent publications [51,52,53,54] or simply using an appending digits mode (NX-2, NX-3, NX-4, NX-5, and NX-6) as was also evident in the literature [25]. Although this is a minor issue, the solution to this problem will promote the smooth development of academic exchanges worldwide. Furthermore, just like the common type B trichothecene producers in FGSC, different chemotypes/genotypes may probably evolve or be identified within the *Fusarium* population in the future.

## 4. Conclusions

Structure differences of trichothecenes resulted from trichothecene biosynthesis gene function diversity. *Tri* genes are usually selected as molecular markers for trichothecene genotyping analysis in *Fusarium* species. In this study, the sequences of the *Tri1* gene from NX-producing strains were compared with common type B trichothecene-producing strains to identify DNA polymorphisms unique to NX producers. Four sets of PCR-RFLP assays were developed by which polymorphisms specific to the *Tri1* gene of NX producers could be rapidly identified to assess their geographical distribution and frequency. The development of high-throughput molecular diagnostic technology for detecting NX-producing FGSC is essential for gathering the primary data on this novel pathogen, such as its geographical distribution, population structure proportion, host range, and other aspects.

## 5. Materials and Methods

### 5.1. Phylogenetic Analysis of Tri1 Gene

Nucleotide sequences of the *Tri1* gene from different *Fusarium* species were retrieved from GenBank and subjected to phylogenetic analysis. In total, 379 *Tri1* gene sequences were analyzed in this work, including 360 sequences from FGSC, 6 type B trichothecene producers from non-FGSC *Fusarium* species, and 13 T-2 *Fusarium* producers. All the *Tri1* genes included in this study can be accessed under the GenBank accession numbers (KX183208–KX183570, KM999941–KM999943, HQ594535–HQ594543, AY040587, GQ915524, JXCE01000207, and GQ915521). Duplicate sequences were removed first before further analyses via multiple sequence alignment, and detailed information for all of the remaining representative sequences can be found in Supplementary Table S1.

Of the 360 FGSC strains, 321 are common type B trichothecene producers, and the remaining 39 are NX producers. These sequences were selected, including FGSC NX-producers and common type B trichothecene producers from different studies [26,27,28]. Following removing duplicate sequences, 88 remained, comprising 6 NX producers and 82 common type B trichothecene producers. The sources, origins, and trichothecene types of these strains can be found in Supplementary Table S1.

In addition to FGSC, other *Fusarium* species could produce common type B trichothecene. Therefore, the *Tri1* gene sequences of the six strains with known trichothecene chemotypes belonging to other *Fusarium* species were also included in this study. These include one *F. culmorum* 3ADON producer (strain 25475, GenBank accession number KX183230), one *F. cerealis* NIV producer (strain 25805, GenBank accession number KX183232), one *F. pseudograminearum* 3ADON producer (strain 28,062, GenBank accession number KX183238), two *F. dactylidis* NIV producers (strains 29,298 and 29,380, GenBank accession number KX183257 and KX183259), and a 3ADON producer 34,461 (GenBank accession number KX183269) with unknown/uncharacterized species. Five sequences remained after duplicate sequences were eliminated (Supplementary Table S1).

In addition, thirteen *Tri1* sequences from *Fusarium* species that produce T-2 toxin were also included in the phylogenetic analyses in this work. The details of these strains include one *F. armeniacum* strain (FRC-R-09335, GenBank accession number GQ915524), one *F. sambucinum* strain (FRC-R-07843, GenBank accession number GQ915521), four *F. langsethiae* strains (NRRL53410, 53417, 53439, Fl201059, GenBank accession numbers HQ594538, HQ594539, HQ594543, and JXCE01000207), two *F. sibiricum* strains (NRRL53421 and 53427, GenBank accession numbers HQ594540 and HQ594541), and five *F. sporotrichioides* strains (NRRL26924, 29977, 29978, 3299, and 53434, GenBank accession numbers HQ594535, HQ594536, HQ594537, AY040587, and HQ594542) [55]. Ten sequences remained after duplicate sequences were eliminated (Supplementary Table S1).

The whole DNA sequences, coding sequences, and gene-encoded amino acid sequences of the *Tri1* gene were submitted to multiple sequence alignment assays to reveal their polymorphisms. Phylogenetic analyses of *Tri1* were conducted under a distance framework using the neighbor-joining algorithm and the Kimura two-parameter model as implemented in MEGA (version 6.0) with the representative sequences. Relative support for individual nodes was assessed via bootstrap analysis with 1000 replications. Moreover, this study determined the potential specific amino acid sites associated with NX or type B trichothecene production using representative amino acid sequences of Tri1.

### 5.2. Primer Design

Specifically, 93 representative *Tri1* gene sequences from different *Fusarium* species were selected for multiple sequence alignment analysis. The species were selected based on their ability to produce either common type B- or NX-trichothecenes, and all the strains included have been previously characterized [27,28]. The alignment allowed designing a common forward primer NX-Tri1F (TGGTCACTAGAATCTCAACACGT) and two reverse primers NX-Tri1R1 (ACTCTCCTGATCTCTTCCTGCA) and NX-Tri1R2 (CACTCGTAGTTGAGCAAAAGGT) according to the conserved sequences of selected *Tri1* gene with the aid of Primer Premier 5 software (PREMIER Biosoft International, San Francisco, CA, USA). The expected amplicon sizes are 439 bp and 899 bp with the primer combinations of Tri1F/R1 and Tri1F/R2, respectively, for all the type B and NX trichothecene-producing FGSC strains.

A PCR-RFLP assay was designed to target variations in the *Tri1* genes that differentiated common type B trichothecenes and NX producers. The DNA fragment obtained with NX-Tri1F/R1 and NX-Tri1F/R2 from the NX strains can be cut into two fragments with different sizes by restriction endonucleases NheI and BclI, respectively, due to several conserved single-nucleotide mutations. However, neither restriction endonucleases can cut the amplicons from all the common type B trichothecene strains, as no corresponding enzyme digestion sites are observed in the nucleotide sequences.

### 5.3. DNA Extraction

*Fusarium* cultures were routinely maintained on Potato Dextrose Agar (PDA) medium for 5 days at 25 °C in the dark. Genomic DNA was extracted using a CTAB-based protocol from lyophilized mycelium and finally dissolved in 100 μL of sterile water as described [21]. In the current study, 30 FGSC strains were tested, and their known trichothecene chemotypes and other detailed information can be found in Supplementary Table S2.

### 5.4. PCR Amplification and Enzyme Digestion

The polymerase chain reaction (PCR) amplifications were performed in a total volume of a 20 μL solution consisting of 1 × T3 Super PCR Mix (Tsingke Biotechnology Co., Ltd., Beijing, China), 0.1 mM of forward (NX-Tri1F) and reverse primers (NX-Tri1R1 or NX-Tri1R2), and approximately 50 ng of DNA template. A negative control without the DNA template was used in every set of reactions. Amplification was carried out in a T100 Thermal Cycler (Bio-Rad, Hercules, CA, USA) programmed for an initial denaturation step at 98 °C for 3 min, followed by 29 cycles of 98 °C for 10 s, 60 °C for 15 s, 72 °C for 15 s, and a final extension of 72 °C for 5 min, according to the manufacturer’s instructions. PCR products were detected via electrophoresis, and the sizes of PCR products were estimated through comparison with DNA standards.

**Figure 7:**
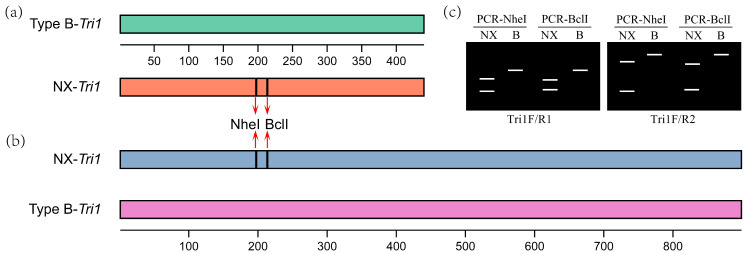
A scheme for the PCR-RFLP analysis of NX producers. (**a**) PCR fragments amplified with primer pair Tri1F/R1 and the positions of NheI and BclI cutting sites; (**b**) PCR fragments amplified with primer pair Tri1F/R2 and the positions of NheI and BclI cutting sites; (**c**) expected electrophoresis patterns of the PCR amplicons after digestion with restriction endonuclease NheI or BclI.

The obtained *Tri1* gene nucleotide fragments were digested with the restriction endonuclease BclI-HF or NheI-HF (New England BioLab Inc., Ipswich, MA, USA). *Tri1* amplicon digestion was performed in a final 20 μL reaction volume. Each reaction comprised 4 μL of amplicon, 2 μL of 10 × rCutSmart buffer, 1 μL of NheI-HF (20 U/μL) or BclI-HF (20 U/μL), and 13 μL of sterile water. The enzyme digestion was performed using a Bio-Rad T100 Thermal Cycler (Hercules, CA, USA) with the following program: 1 digestion cycle at 37 °C for 2 h. As previously mentioned, the digested PCR products were separated via electrophoresis, and different band patterns (numbers and sizes) will be observed for NX and type B trichothecene producers. A scheme for the PCR-RFLP analysis is shown in Figure 7.

## Data Availability

Data sharing is not applicable to this article.

## Supplementary Materials

The following supporting information can be downloaded at: https://www.mdpi.com/article/10.3390/toxins15120692/s1, Table S1: the sources, origins, and trichothecene types of the 103 representative *Fusarium* strains; Table S2: the thirty FGSC strains tested in this study.
